# Supplementary material for: Identification of Freezing-Responsive microRNAs and Their Targets in Chinese Jujube by Small RNA and Degradome Sequencing
Source: Int J Mol Sci. 2026 Sep 4;27(17):7912. doi: 10.3390/ijms27177912 (PMC13566890; doi:10.3390/ijms27177912)
Supplement: Supplementary file 1 [file ijms-27-07912-s001.zip › Supplementary Material S2/novel_miR_60.pdf]

Provisional ID	:	novel_miR_60
Score total	:	0.7
Score for star read(s)	:	-1.3
Score for read counts	:	0
Score for mfe	:	0.4
Score for randfold	:	1.6
Score for cons. seed	:	
Total read count	:	433
Mature read count	:	278
Loop read count	:	2
Star read count	:	153

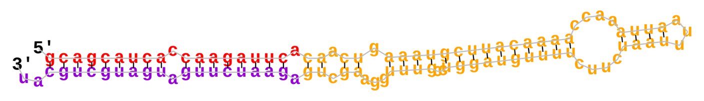

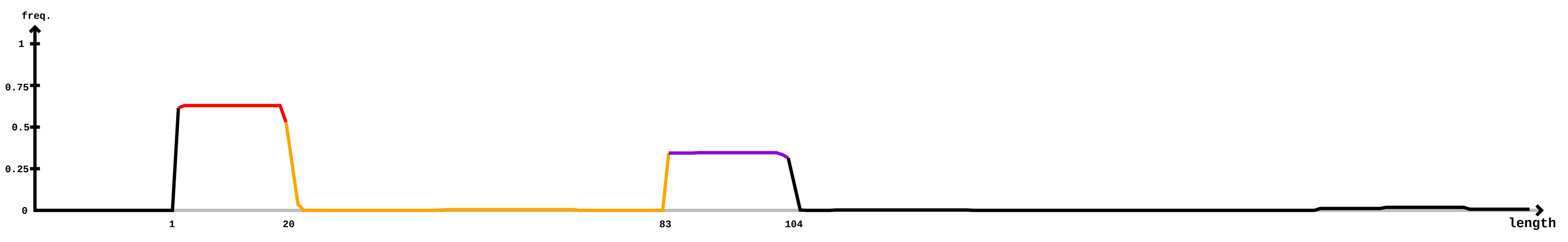

Mature **Star**

```
5'- augagagucauuguuugcaggugcagcaucaccaagauucacaacugaaaugcuuacaaaaccaaauuaauuuaaucuucuuuuguagguccguuuggaagcugagaaucuugaugaugcugcaucagccauagaugacuguauucacccaaagcccaccauauuuguuuugaagauucacauccgaucaaagauuauacauacuauucguuuauuguuucuuuucuuguauaacacugauuugcuuugu -3'  obs
    augagagucauuguuugcaggugcagcaucaccaagauucacaacugaaaugcuuacaaaaccaaauuaauuuaaucuucuuuuguagguccguuuggaagcugagaaucuugaugaugcugcaucagccauagaugacuguauucacccaaagcccaccauauuuguuuugaagauucacauccgaucaaagauuauacauacuauucguuuauuguuucuuuucuuguauaacacugauuugcuuugu       exp
    .((((((((((..(.((.((((((((((((.((((((((.((.((.((((((((((((((....((((...))))....))))))))))..))))...)).)).))))))))).))))))))))))).)).)..)))))))...))))..((((((...............(((....))).....(((((..(.(((((((.......................))))))))..))))).)))))).
```

read	reads	mm	sample
gcagcaucaccaagauuA	25	1	LCK-2
gcagcaucaccaagauuA	1	1	LT-1
gcagcaucaccaagauuc	1	0	JCK-1
gcagcaucaccaagauuA	17	1	LCK-1
gcagcaucaccaagauuca	41	0	LCK-3
Acagcaucaccaagauuca	1	1	LCK-3
gcagcaucaccaagauuca	13	0	JCK-3
gcagcaucaccaagauuca	5	0	JT-2
gcagcaucaccaagauuca	10	0	JT-3
gcagcaucaccaagauuca	34	0	LT-2
gcagcaucaccaagauuca	16	0	JCK-1
gcagcaucaccaagauuca	28	0	LT-1
gcagcaucaccaagauuca	18	0	JCK-2
gcagcaucaccaagauuca	33	0	LT-3
gcagcaucaAcaagauuca	1	1	LT-1
gcagcaucaccaagauucac	4	0	LT-3
gcagcaucaccaagauucac	3	0	JCK-3
gcagcaucaccaagauucac	3	0	JCK-2
gcagcaucaccaagauucac	5	0	JCK-1
gcagcaucaccaagauucaca	1	0	JCK-1
gcagcaucaccaagauucaca	4	0	LT-1
gcagcaucaccaagauucaca	3	0	JCK-3
gcagcaucaccaagauucaca	5	0	JCK-2
cagcaucaccaagauucac	1	0	JCK-2
cagcaucaccaagauucac	2	0	LT-1
cagcaucaccaagauucacU	2	1	JCK-1
cagcaucaccaagauucacU	1	1	JCK-2
uuaauuuaaucuuUuuuuguagg	1	1	LT-3
uaauuuaaucuuUuuuuguagg	1	1	LT-1
agaaucuugaugaugcugc	4	0	LT-1
agaaucuugaugaugcugc	1	0	LT-3
agaaucuugaugaugcugca	1	0	JCK-2
agaaucuugaugaugcugca	4	0	LT-2

Mature Star

augagagucauuguuugcaggugcagcaucaccaagauucacaacugaaaugcuuacaaaaccaaauuaauuuaaucuucuuuuguagguccguuuggaagcugagaaucuugaugaugcugcaucagccauagaugacuguauucacccaaagcccaccauauuuguuuugaagauucacauccgaucaaagauuauacauacuauucguuuauuguuucuuuucuuguauaacacugauuugcuuugu			
....agaaucuugaugaugcugca..	4	0	LT-1
....agaCucuugaugaugcugcau...	1	1	LT-2
....agaaucuugaugaugcugcau...	15	0	JCK-3
....agaaucuugaugaugcugcau...	26	0	JCK-2
....agaaucuugaugCugcugcau...	1	1	JCK-2
....agaaucuugaugaugcugcaC...	2	1	JCK-1
....agaaucuugaugaugcugcaC...	1	1	JCK-2
....agaaucuugauUaugcugcau...	1	1	JCK-3
....agaaucuugaugaugcugcaC...	1	1	LT-3
....agaaucuugaugaugcugcau...	19	0	LT-2
....agaaucuugaugaugcugcau...	13	0	JCK-1
....agaaucuugaugaugcugcau...	6	0	LCK-3
....agaaucuugaugaugcugcau...	27	0	LT-1
....agaaucuugaugaugcugcaA...	1	1	JCK-2
....agaCucuugaugaugcugcau...	1	1	JCK-2
....agaaucuugaugaugcugcau...	19	0	LT-3
....agaauUuugaugaugcugcau...	2	1	JCK-3
....agaaucuugaugaugcugcauU..	2	1	JCK-2
....cuugaugaugcugcauca...	1	0	LCK-3
....agaugacuguauucacccaaagc...	1	0	JCK-2
....auuguuucuuuucuuguauUacacu............	2	1	JCK-3
....auuguuucuuuucuuguauUacacu............	3	1	JCK-1
....ucuuguauaacacugauuugcuuug.	3	0	LT-1
